# New-onset graves’ disease after the initiation of nivolumab therapy for gastric cancer: a case report

**DOI:** 10.1186/s12902-020-00613-5

**Published:** 2020-08-26

**Authors:** Hiroshi Yamada, Fumitaka Okajima, Takeshi Onda, Shunji Fujimori, Naoya Emoto, Hitoshi Sugihara

**Affiliations:** 1 Department of Endocrinology, Diabetes and Metabolism, Graduate School of Medicine, Nippon Medical School, 1-1-5, Sendagi, Bunkyo-ku, Tokyo, 113-8603 Japan; 2 Department of Gastroenterology, Nippon Medical School Chiba Hokusoh Hospital, 1715, Kamagari, Inzai, Chiba, 270-1694 Japan

**Keywords:** Graves’ disease, Nivolumab, Thyrotoxicosis, Immune checkpoint inhibitor, ^99m^Tc-pertechnetate scintigraphy, Thyroid-stimulating hormone receptor antibody

## Abstract

**Background:**

Immune checkpoint inhibitors (ICIs) can induce immune-related adverse events (irAEs) including thyroid dysfunction. There are only a few reports on Graves’ disease induced by ICIs. We report a case of new-onset Graves’ disease after the initiation of nivolumab therapy in a patient receiving gastric cancer treatment.

**Case presentation:**

The patient was a 66-year-old Japanese man, who was administered nivolumab (240 mg every 3 weeks) as a third-line therapy for stage IVb gastric cancer. His thyroid function was normal before the initiation of nivolumab therapy. However, he developed thyrotoxicosis before the third administration of nivolumab. Elevated, bilateral, and diffuse uptake of radioactive tracer was observed in the ^99m^Tc-pertechnetate scintigraphy. Furthermore, the thyroid-stimulating hormone receptor antibody (TRAb) and thyroid-stimulating antibody (TSAb) test results, which were negative before the first administration of nivolumab, were positive after starting the therapy. The patient was diagnosed with Graves’ disease, and the treatment with methimazole and potassium iodide restored thyroid function.

**Conclusions:**

This is the first complete report of a case of new-onset Graves’ disease after starting nivolumab therapy, confirmed by diffusely increased thyroid uptake in scintigraphy and the positive conversion of antibodies against thyroid-stimulating hormone receptor. It is important to perform thyroid scintigraphy and ultrasonography to accurately diagnose and treat ICI-induced thyrotoxicosis, because there are various cases in which Graves’ disease is developed with negative and positive TRAb titres.

## Background

Immune checkpoint inhibitors (ICIs), such as cytotoxic T-lymphocyte-associated protein 4 (CTLA-4), programmed cell death protein-1 (PD-1), and programmed death ligand 1 (PD-L1) inhibitors, have been widely used as a standard cancer treatment during recent years. However, occasionally, ICIs cause immune-related adverse events (irAEs), which affect different organs, such as the lung, gastrointestinal tract, liver, nervous system, skin, and endocrine glands. The endocrine irAEs include hypophysitis, thyroid dysfunction, adrenal insufficiency, and type 1 diabetes. While endocrine irAEs due to CTLA-4 inhibitors, such as ipilimumab and tremelimumab, mainly include pituitary dysfunction, those due to PD-1 inhibitors, such as nivolumab and pembrolizumab, are mainly related to thyroid dysfunction [1–4]. The PD-1 inhibitor-induced thyroid dysfunction often includes hypothyroidism rather than hyperthyroidism [1, 2, 5, 6]. Thyrotoxicosis following ICI therapy is caused mostly by thyroiditis syndrome, which has been reported to spontaneously recover with the subsequent hypothyroidism in many cases [2, 7, 8]. However, Graves’ disease induced by ICI treatment has not been extensively explored. Here, we present a case of Graves’ disease shortly after the initiation of nivolumab therapy for gastric cancer.

## Case presentation

A 66-year-old man was diagnosed with stage IVb (T4bN0M1) human epidermal growth factor receptor 2 (HER2)-positive gastric cancer at Nippon Medical School Chiba Hokusoh Hospital, one and a half years before the onset of thyrotoxicosis. After diagnosis, he was not referred for surgery because of liver metastasis with a portal tumour thrombus; rather, the patient received 8 cycles of first line chemotherapy with a combination of tegafur/gimeracil/oteracil (S-1), cisplatin, and trastuzumab. However, the patient presented with progressive disease, assessed based on the computed tomography (CT) and oesophagogastroduodenoscopy (OGD) evaluations following the first line therapy. Hence, he received a second line chemotherapy with paclitaxel and ramucirumab. After 4 cycles of the second line chemotherapy, although there was a reduction in tumour size, after 10 cycles, the patient presented with progressive disease, as assessed by CT. At this stage, nivolumab (240 mg every 3 weeks) was started. The patient had a normal thyroid function before the first administration. However, TSH suppression was observed before the second administration, and thyrotoxicosis occurred before the third administration of the drug; hence, nivolumab therapy was discontinued and the patient was referred to our department.

**Fig. 1:**
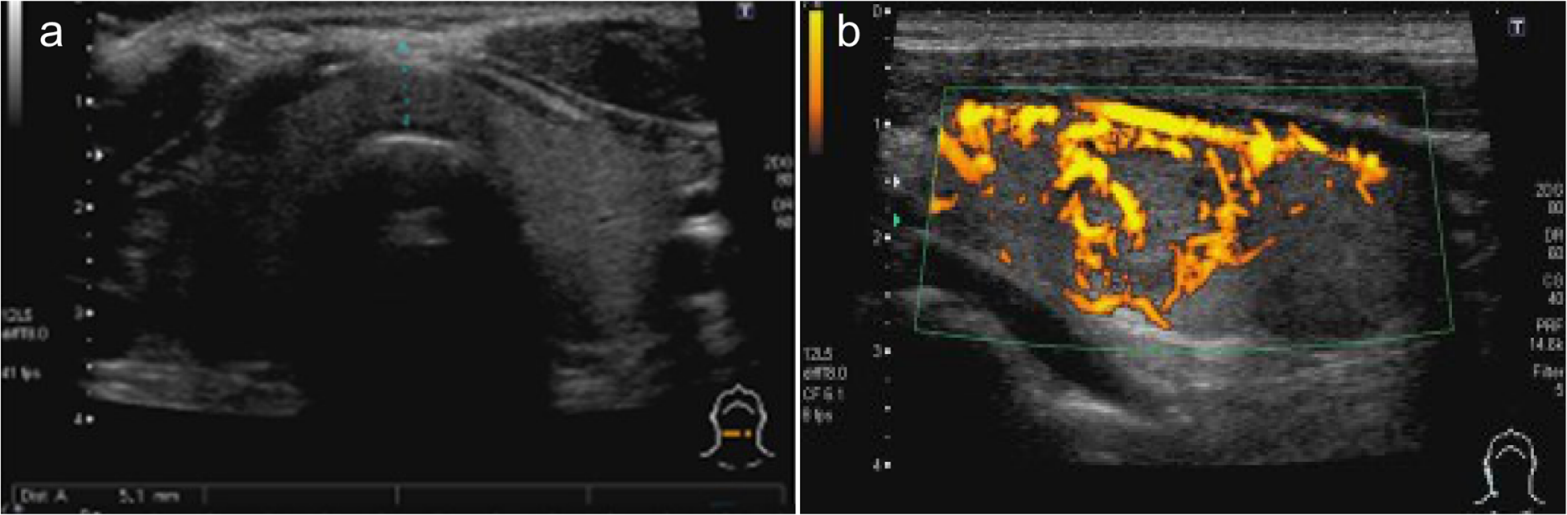
Thyroid ultrasonography of the patient. **a** Slight swelling in isthmus. **b** Rich blood flow in parenchyma

**Fig. 2:**
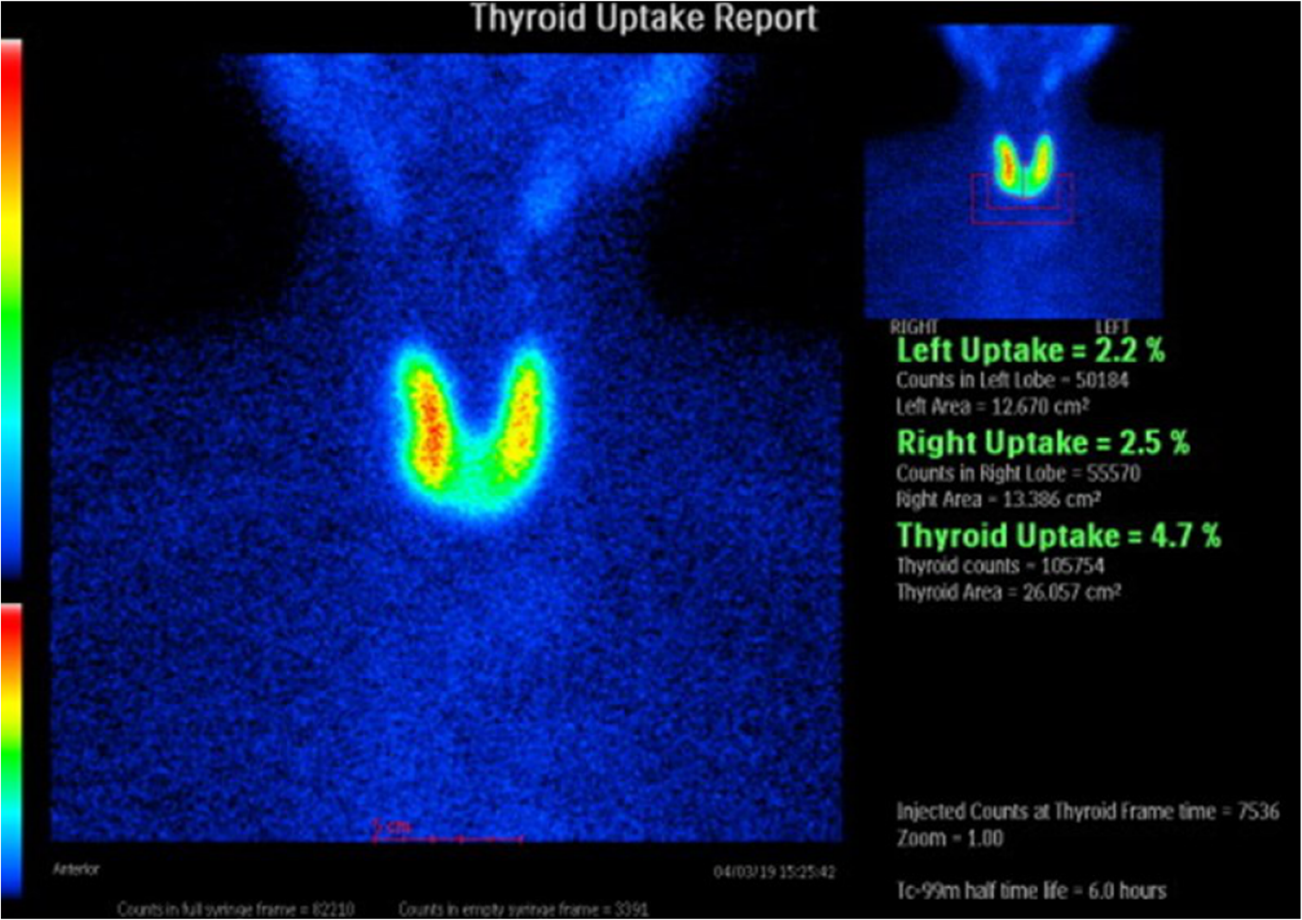
^99m^Tc-pertechnetate scintigraphy showing elevated, bilateral, and diffuse uptake of the radioactive tracer (4.7%)

The patient had complained of fatigue and shortness of breath during exertion. His height was 174.5 cm, body weight was 79.85 kg, heart rate was 114 beats per minute, and blood pressure was 86/62 mmHg. There was no evidence of Graves’ orbitopathy or pretibial myxedema. He and his family members had no history of thyroid diseases. The thyroid-stimulating hormone (TSH), free triiodothyronine (FT3), and free thyroxine (FT4) levels were < 0.010 μIU/mL, 15.30 pg/mL, and > 5.00 ng/dL, respectively (Table 1). The titres of thyroid-stimulating hormone receptor antibody (TRAb) and thyroid-stimulating antibody (TSAb) were positive (24.2 IU/L and 2184%, respectively), whereas those of anti-thyroglobulin antibody (TgAb) and anti-thyroid-peroxidase antibody (TPOAb) were negative (14.1 IU/L and < 9.0 IU/L, respectively). The thyroglobulin (Tg) level was 347.00 ng/mL. Thyroid ultrasonography showed slight goitre (Fig. 1a) and rich blood flow in the parenchyma (Fig. 1b). ^99m^Tc-pertechnetate scintigraphy, which was performed on the first consultation day of the patient at our department, showed elevated, bilateral, and diffuse uptake of the radioactive tracer (Fig. 2). We measured anti-thyroid autoantibodies in preserved serum samples. The titres of TRAb and TSAb were negative before the first administration of nivolumab, whereas they were positive (3.1 IU/L and 227%, respectively) before the second administration. Thus, we diagnosed his thyrotoxicosis as new-onset Graves’ disease after the initiation of nivolumab therapy. The human leukocyte antigen (HLA) typing of the patient showed the following allelic variants: A*24:02/*26:01*, B*51:01/*54:01*, C*01:02/*15:02*, DRB1*04:05/*15:01*, DQA1*01:02/*03:01*, DQB1*04:01/*06:02*, DPA1*02:02, and DPB1*05:01.

**Table 1:** TSH, FT3, FT4, and Tg levels and TRAb and TSAb titres in our patient

	Day of nivolumab administration								
	0	21	43	72	100	107	114	121	139
TSH (μIU/mL)	2.822	0.133	< 0.010	< 0.010	< 0.010	< 0.010	0.027	0.034	0.010
FT3 (pg/mL)	1.73	2.82	15.30	2.57	1.80	< 1.50	1.98	1.58	1.67
FT4 (ng/dL)	0.86	1.15	> 5.00	1.18	0.82	0.80	0.70	0.70	1.18
TRAb (IU/L)	< 1.0	3.1	24.2	27.5	NA	NA	NA	NA	10.7
TSAb (%)	95	227	2184	NA	NA	1683	NA	NA	667
Tg (ng/mL)	11.20	40.70	347.00						28.70

Day 0: first administration of nivolumab, Day 21: second administration of nivolumab

The normal range of the thyroid parameters is as follows: TSH (0.350–4.940 μIU/mL), FT3 (1.88–3.18 pg/mL), FT4 (0.70–1.48 ng/dL), TRAb (< 1.0 IU/L), TSAb (≤ 120%), and Tg (≤ 33.70 ng/mL)

**Fig. 3:**
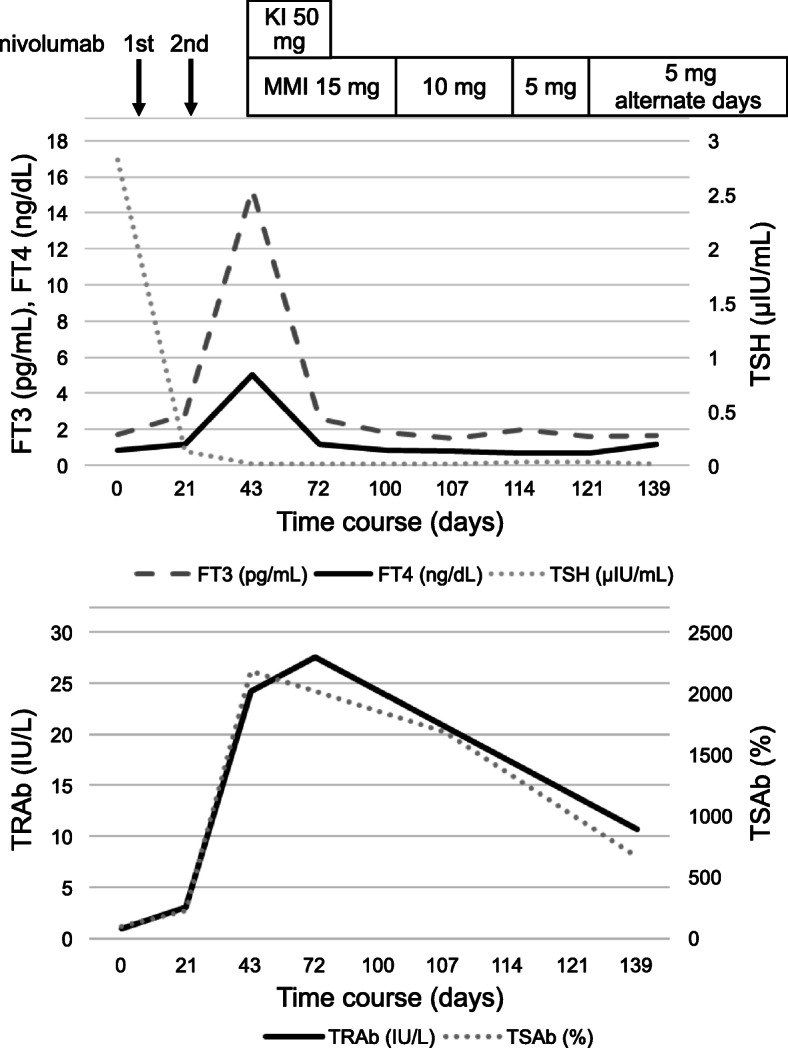
Clinical course of the patient. MMI: methimazole, KI: potassium iodide. Day 0: first administration of nivolumab, Day 21: second administration of nivolumab

We treated the patient with methimazole (MMI) at a dose of 15 mg/day and potassium iodide (KI) at a dose of 50 mg/day. One month after the initiation of the therapy, when the FT3 and FT4 levels of the patient were normal, we discontinued KI. Gradually, we reduced the dosage of MMI, and the continued administration (till the death of the patient) of MMI at a dose of 5 mg every alternate day stabilised his thyroid function (Fig. 3).

Furthermore, as nivolumab was found to be ineffective (based on the CT and OGD evaluations), the patient received irinotecan therapy. However, after 2 cycles of chemotherapy, the patient was diagnosed with brain metastasis by magnetic resonance imaging (MRI), for which, he received gamma knife and steroid therapy. The patient died 4 months after his first visit to our department.

## Discussion and conclusions

We present a case of new-onset Graves’ disease after the initiation of nivolumab therapy in a patient receiving gastric cancer treatment. Thyrotoxicosis, induced by ICIs, is mainly a form of destructive thyroiditis. Three cases of new-onset Graves’ disease during nivolumab therapy, other than the present case, have been reported [9–11] (Table 2). Iadarola et al. [9] reported a case of Graves’ disease-like hyperthyroidism after the second administration of nivolumab in a patient with left lung carcinoma. In this case, ^99m^Tc-pertechnetate scintigraphy in the patient with T3-toxicosis showed diffuse thyroid uptake of the radionuclide suggesting Graves’ disease-like hyperthyroidism, whereas the TRAb tests were consistently negative. Thyroid ultrasonography showed a multinodular goitre, with a normo-echoic pattern and normal vascularity of the parenchyma [9]. Brancatella et al. [10] reported a case similar to that of Iadarola et al. [9] with diffuse thyroid uptake and negative TRAb titre. In this case, ultrasonography showed an enlargement of the thyroid with a hypoechoic pattern and mild hypervascularity. Kurihara et al. [11] reported a case of simultaneous development of Graves’ disease and type 1 diabetes mellitus during nivolumab therapy. In this case, thyrotoxicosis was detected after the sixth administration of nivolumab, with positive TRAb titre. However, ultrasonography showed no enlargement of the thyroid and a normal vascularisation pattern. This patient was clinically diagnosed as mild Graves’ disease and treated with MMI [11]. Unlike these cases, our case is important in terms of confirmation of both positive TRAb titre and diffuse thyroid uptake in scintigraphy. Moreover, titres of TRAb and TSAb were converted from negative to positive after starting nivolumab therapy. It seems reasonable to presume that Graves’ disease was induced by nivolumab, although there is a possibility of coincidence. Furthermore, our patient had HLA-DPB1*05:01, which has been reported to be associated with Japanese Graves’ disease [12, 13]. Although the involvement of HLA cannot be argued based only on a single case, accumulating similar cases might help clarify the mechanism of development of rare ICI-induced Graves’ disease.

**Table 2:** Comparison of case reports on new-onset Graves’ disease during nivolumab therapy

Study	TSH (μIU/mL)	FT3 (pg/mL)	FT4 (ng/dL)	TRAb (IU/L)		TSAb (%)		US	RAIU/ ^99m^Tc uptake	HLA
				Before	After	Before	After			
Iadarola et al. [9]	< 0.01	5.71	1.36	NA	Negative	NA	NA	Normal	High (^99m^Tc)	NA
Brancatella et al. [10]	0.04	7.29	2.28	NA	Negative	NA	NA	Hyper-vascular	High (RAIU)	NA
Kurihara et al. [11]	0.008	3.88	1.72	NA	Positive (3.7)	NA	NA	Normal	NA	DRB1*04:05
Yamada et al. (present case)	< 0.010	15.30	> 5.00	Negative (< 1.0)	Positive (24.2)	Negative (95)	Positive (2184)	Hyper-vascular	High (^99m^Tc)	DPB1*05:01

*US* ultrasonography, *RAIU* radioactive iodine uptake, Before: before the initiation of nivolumab therapy, After: after the initiation of nivolumab therapy (at the onset of the thyrotoxicosis)

Graves’ disease induced by ICIs other than nivolumab has been rarely reported. Azmat et al. [14] reported a case of ipilimumab-induced thyrotoxicosis caused by Graves’ disease. Gan et al. [15] reported a case of tremelimumab-induced Graves’ hyperthyroidism. Yajima et al. [16] reported a case of Graves’ disease induced by pembrolizumab, a PD-1 inhibitor. In this case, TRAb was positive after the fifth administration of pembrolizumab, and thyroid ultrasonography showed a mild increase in the intra-thyroidal blood flow. A thyroid scintigraphy was not performed because of the iodine treatment [16]. The cases of nivolumab-induced Graves’ disease with negative TRAb titre suggest that performing thyroid scintigraphy and ultrasonography can help to accurately diagnose and treat ICI-induced thyrotoxicosis.

A relationship between thyroid antibodies and PD-1 inhibitor-induced thyroid dysfunction has not been explained. Kimbara et al. [8] suggested that patients with pre-existing TgAb and an elevated TSH level at baseline are at a higher risk of thyroid dysfunction induced by nivolumab. Osorio et al. [17] reported an association between positive thyroid antibodies (anti-thyroglobulin or anti-microsomal antibodies) and thyroid dysfunction induced by ICIs. In the studies on new-onset Graves’ disease during nivolumab therapy, it is interesting to note that in two caucasian patients with Graves’ disease, TRAb was negative [9, 10]. Furthermore, TRAb was positive in two Japanese patients, including our patient [11] (Table 2). However, additional evidence is required to reveal the role of TRAb in the pathogenesis of ICI-induced hyperthyroidism.

A limitation of our case was radioactive iodine uptake (RAIU) was not performed. Because imaging with ^99m^Tc-pertechnetate reflects both blood flow and uptake via the symporter and does not assess organification, malignant nodules may appear hyperfunctioning in pertechnetate imaging, but hypofunctioning in ^123^I-imaging. In our study, tumours were not detected although a part of ^99m^Tc uptake was stronger.

In conclusion, we reported a case of Graves’ disease shortly after the initiation of nivolumab therapy for gastric cancer. Our case presented a typical Graves’ disease with both positive TRAb titre and diffuse thyroid uptake in scintigraphy. Moreover, our case is valuable in terms of confirming the conversion of TRAb and TSAb from negative to positive titres after starting the therapy. It is important to perform thyroid scintigraphy and ultrasonography because there are cases of nivolumab-induced Graves’ disease with negative TRAb titre as previously reported. To reveal the pathogenesis of ICI-induced Graves’ disease, it is necessary to study additional cases of similar nature.

## Data Availability

The data that support the findings of this study are stored in Nippon Medical School Chiba Hokusoh Hospital (Inzai, Chiba) and available from the corresponding author on reasonable request.

## Abbreviations

CT: Computed tomography
CTLA-4: Cytotoxic T-lymphocyte-associated protein 4
FT3: Free triiodothyronine
FT4: Free thyroxine
HER2: Human epidermal growth factor receptor 2
HLA: Human leukocyte antigen
ICI: Immune checkpoint inhibitor
IrAE: Immune-related adverse event
KI: Potassium iodide
MMI: Methimazole
MRI: Magnetic resonance imaging
OGD: Oesophagogastroduodenoscopy
PD-1: Programmed cell death protein-1
PD-L1: Programmed death ligand 1
RAIU: Radioactive iodine uptake
Tg: Thyroglobulin
TgAb: Anti-thyroglobulin antibody
TPOAb: Anti-thyroid-peroxidase antibody
TRAb: TSH receptor antibody
TSAb: Thyroid-stimulating antibody
TSH: Thyroid-stimulating hormone
